# Presentation, management and outcome of thoracic trauma in a resource-limited environment: A case series

**DOI:** 10.1016/j.tcr.2025.101155

**Published:** 2025-02-28

**Authors:** Danielle Benjamin, Patrick Charlorin, Gérald Jonacé, Jude Milcé

**Affiliations:** a State University of Haiti, Faculty Of Medicine and Pharmacy, Port-au-Prince, Ouest, Haiti; b Hospital of the State University of Haiti, Thoracic And Vascular Surgery, Port-au-Prince, Ouest, Haiti; c Ephata Medical Complex, General Surgery, Port-au-Prince, Ouest, Haiti

**Keywords:** Thoracic trauma, Chest trauma, Case series, Lower-middle-income country, Haiti, HUEH, Thoracic surgery

## Abstract

**Background:**

Trauma is a major public health problem, causing the death of >5 million people each year. One-fifth of these deaths are related to thoracic trauma (TT). This study aims to provide data on the presentation, management, and outcome of TT at the State University Hospital of Haiti (HUEH).

**Methods:**

This is a retrospective, single-center, formal case series of 35 cases of TT admitted to the General Surgery Department of HUEH from January 2013 to December 2017. Data analyzed included sociodemographic, preoperative (etiology and clinical presentation), management, and outcomes. The Exact Fischer, Welch and Mann-Whitney *U* tests were used. A *P*-value *p* < 0.05 was considered significant. The case series was reported according to PROCESS criteria.

**Results:**

Of our sample of 35 patients, there was a male predominance (*n* = 27, 77.1 %), and the median age was 40 years. Most patients (*n* = 33, 94.3 %) presented with penetrating TT. The principal etiology was assaults (*n* = 30, 85.7 %), committed mostly by strangers (*n* = 19) and mainly with firearms (*n* = 18). Topping the list of common thoracic injuries were hemopneumothorax, diaphragmatic tear and open pneumothorax. There were extrathoracic associated injuries in 16 patients (45.7 %) with abdominal involvement in 10 cases (28.6 %). Principal specific management was tube thoracostomy for 33 patients (94.3 %) with additional laparotomy for 12 patients (34.3 %) for a median hospital stay of 6 days. There were 2 deaths due to massive hemorrhage without the possibility of massive transfusion. Estimated blood loss was significantly related to short-term survival outcome (*p* = 0.02).

**Conclusion:**

This is a rare study in the Haitian environment that explores TT. Rather than road traffic accidents, assaults caused mainly by firearms were the chief cause of TT and testify to the current climate of violence and insecurity in the country. Although most of the TT cases were manage by tube thoracostomy, one third needed additional laparotomy. Increased blood loss associated with poorer patient outcomes highlights the need for more transfusion services and the establishment of standard of care for TT in Haiti.

## Highlights

• Assaults by firearms are the chief cause of thoracic trauma at HUEH, testifying to the current violent climate in Haiti.
• Estimated blood loss is associated with poorer patient outcomes highlighting the problem in transfusion services in Haiti.
• There is a need to establish a trauma registry and to standardize the management of thoracic trauma in Haiti.

## Introduction

Trauma is considered a major public health problem, according to the World Health Organization (WHO), causing >5 million deaths annually [1]. One fifth of these deaths are related to thoracic trauma (TT). Considering the mechanism, TT are classified as either blunt or penetrating. The first, caused mainly by road traffic accidents (RTA) is the most frequent in developed countries, representing up to 80 %; while the latter represents around 20 % of the TT and caused either by gunshot wound (GSW) or penetrating stab wounds (PSW) [2].

In lower-middle-income-countries (LMIC) the burden of trauma is heavier because of many reasons including the lack of trauma registry, a tool used by developed countries to improve trauma care [3]. Haiti is a LMIC, the poorest of the American continent, that has been facing a worsening of its situation after a devastating earthquake in 2010 [4]. That impoverishment is leading to road and physical insecurity [5]. The State University Hospital of Haiti (HUEH), commonly known as general Hospital, is the largest public hospital, and it's located in the capital Port-au-Prince. It has been destroyed after the earthquake of 2010 and at the time of our study, most patients were admitted in temporary shelters as the new 534-bed facility was still under construction [6]. Even if it's a tertiary facility, there isn't a thoracic surgery department nor an intensive care unit (ICU), and the TT cases are taken care in the general surgery unit after triage in the emergency department.

Currently, little is known in the scientific literature about TT in Haiti. This article aims to be one of the first to provide data on the presentation, management, and outcome of chest trauma in Haiti. In a global surgery era, such scientific information on health challenges related to surgical care, particularly in vulnerable populations, should be made available to the scientific community in order to expand the provision of care for attaining better health for all.

## Materials and methods

### Study design

This formal case series is a descriptive and monocentric study with a retrospective collection of data over a period of five years (from January 1st 2013 to December 31st 2017), in the general surgery department of HUEH.

### Inclusion and exclusion criteria

Using the hospital's main logbook, the surgical unit registry, and the patients' medical charts, the subjects were selected based on the most complete charts we found. Patients included in our study were first identified upon the diagnosis of TT, and then their respective medical records, when found, were extracted to confirm the diagnosis and for further data collection. We excluded the pediatric population and all patients who were admitted and discharged from the emergency department. We also excluded all patients who were admitted in a state of cardiac arrest or found dead.

### Ethical approval

This study was approved by HUEH administration and ethical clearance for the research was obtained from the LABMES (Laboratory of Medicine Ethics and Society), which is the institutional ethics committee attached to the Faculty of Medicine of The State University of Haiti.

### Data collection and procedure

Information sought included socio-demographic, preoperative, management, and postoperative. It was collected on a Microsoft excel database. The socio-demographic data included age, sex, hospital admission time. The preoperative data included etiology, mechanism of injury, clinical presentation, laboratory value and imaging. Management data included pain control, antibiotic therapy, surgical care, intraoperative findings, estimated blood loss (EBL), and transfusion. Postoperative data included length of hospitalization, and outcome such as complication and death.

### Statistical methods

The data were collected and entered into an excel sheet, after coding. They were transferred to the statistical software PVALUE.IO and R studio version 4.4.1 [7]. Descriptive statistics included median [min max] for continuous variables and proportion (percentage) to describe categorial variables. Association among various study parameters were assessed using the Exact Fischer-test, Welch, and Mann-Whitney *U* test. Statistical significance was assumed at the *p* < 0.05 level.

## Results

### Sociodemographic characteristics

A total of 35 patients met our inclusion criteria. There were 27 males (77.1 %) and 8 females (22.9 %) with a male-female sex ratio of 3:1. The median age was 40 years with age range from 21 to 64 years. Majority of patients (*n* = 16, 45.7 %) were in age group 31 to 50 years. Median hospital admission time was 6 h, range from 0.5 h to 24 h; with 5 patients (14.3 %) arriving within one hour, and 16 patients (45.7 %) arriving between 1 and 6 h (Table 1).

**Table 1:** Demographic, clinical and paraclinical characteristics of the study patients.

	N	%	Median[q75-q25]	Min	Max
Sex					
Male	27.0	77.14			
Female	8.0	22.86			
Age (year)	35.0		40.0 [26.0; 47.0]	21.0	64.0
Male	27.0		40.0[26.0; 46.0]	21.0	60.0
Female	8.0		37.0[22.8; 48.2]	21.0	64.0
<31	15.0	43.0			
31–50	16.0	46.0			
51–70	4.0	11.0			
Hospital admission time (hour)	32.0		6.00 [2.50; 12.0]	0.5	24.0
<1	5.0	14.3			
1–6	16.0	45.7			
7–24	11.0	31.4			

Vital signs at admission					
SBP (mmHg)	32.0		100 [97.5; 122]	70.0	180
Heart rate	32.0		95.5 [85.5; 104]	60.0	140.0
Tachycardia	11.0	31.4			
Respiratory rate	32.0		32.0 [28.0; 37.2]	24.0	52.0
Temperature	30.0		36.4 [36.1; 36.8]	34.0	38.0
Hypothermia	3.0	8.6			

Mechanism of injury					
Penetrating	33.0	94.0			
Blunt	2.0	6.0			

Affected side					
Left hemithorax	20.0	57.0			
Right hemithorax	11.0	32.0			
Bilateral	4.0	11.0			

Etiology					
Assault Gunshot wound (18.0,60.0) Penetrating stab wound (12.0,40.0)	30.0	85.7			
Road traffic accident	2.0	5.7			
Fall	2.0	5.7			

Authors of assaults					
Strangers	19.0	73.0			
Police officers	2.0	8.0			
Relatives	4.0	15.0			
Suicide attempt	1.0	4.0			

Laboratory					
Hematocrit	28.0		34.0[26.8;39.0]	6.0	42.0
Hemoglobin	28.0		11.3[8.9;12.9]	2.0	13.0
Severe anemia	5.0	14.0			

ASA score					
4E^a^	2.0	5.7			

Imaging study					
Xray	6.0	17.1			
FAST	1.0	2.9			

a E = emergency.

### Preoperative data

#### Etiology and clinical presentation

Etiology and clinical presentation are detailed in Table 1. The most frequent etiology was assault sustained by 30 patients (85.7 %) while the rest sustained RTA and fall. Among the assaulted, 18 were GSW (51.4 %) and 12 PSW (34.3 %). For 26 patients, the authors of the assaults were identified. When that information was available in the charts, the attacks were mostly committed by strangers in 19 cases (54.3 %), followed by relatives in 4 cases (11.4 %), and police officers in two cases (5.7 %). One case of PSW was an attempted suicide. In the RTA group, all the cases were pedestrians hit by moving vehicles.

The predominant mechanism of injury was penetrating thoracic trauma encountered in 33 patients (94.3 %) whereas the remaining was blunt trauma. Most of the injuries occurred on the left side (*n* = 20, 57.1 %), while 11 occurred on the right side (31.4 %) and the remaining 4, had a bilateral TT (11.4 %). Concerning the vital signs, the median SBP was 100 mmHg, range from 70 mmHg to 180 mmHg and 25 % of our patients arrived with a systolic blood pressure (SBP) <97.5 mmHg. There were 11 patients (31.4 %) with tachycardia (HR > 100) and all our patients presented tachypnea. The median temperature was 36.4 degrees Celsius with 2 patients presenting hypothermia.

#### Laboratory and imaging

Laboratory findings are detailed in Table 1. The median hemoglobin level was 11.3 g/dl (hematocrit =34), ranging from 2 g/dl (hematocrit =6) to 13.9 g/dl (hematocrit =42). Five patients came with severe anemia (14.3 %). As for imaging, chest x-ray was available for 6 patients (17.1 %), one patient had a Focus Assessment with Sonography for Trauma (FAST) beside the X-ray, none had a CT-scan.

### Management and intraoperative findings

**Fig. 1:**
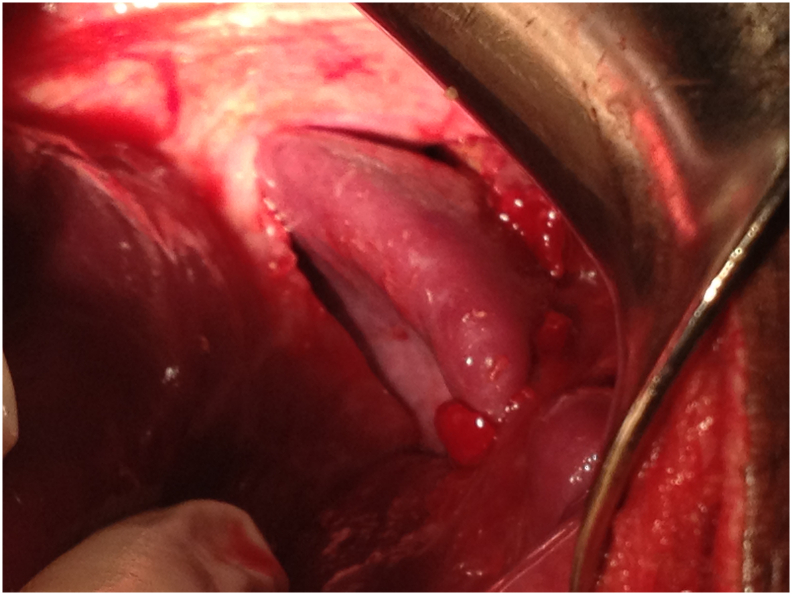
Intraoperative abdominal view of the left hemidiaphragm showing the left lung protruding through a diaphragmatic tear during a laparotomy for thoracoabdominal gunshot wound.

Injury assessment showed that the most common thoracic injuries by order of frequency were hemopneumothorax (*n* = 14, 40 %), followed by diaphragmatic tear (*n* = 9, 25.7 %), open pneumothorax (*n* = 7, 20 %), minim hemothorax (n = 7, 20 %), simple pneumothorax (*n* = 5, 14.3 %), rib fractures (*n* = 2, 5.7 %), massive hemothorax (n = 2, 5.7 %) and pulmonary contusion (n = 1, 2.9 %) (Fig. 1, Fig. 3).

Besides the TT, 16 patients (45.7 %) presented at least one associated injury and 5 among them (14.3 %) sustained two associated injuries. When an associated injury was present, the most frequent was abdominal injury (*n* = 10, 28.6 %), followed by injury of the extremities (*n* = 8, 22.9 %), and spinal cord injury (*n* = 3, 8.6 %). The preoperative physical status classification of the American Society of Anesthesiologists showed two patients (5.7 %) with a score ASA-IVE (Table 1).

**Fig. 2:**
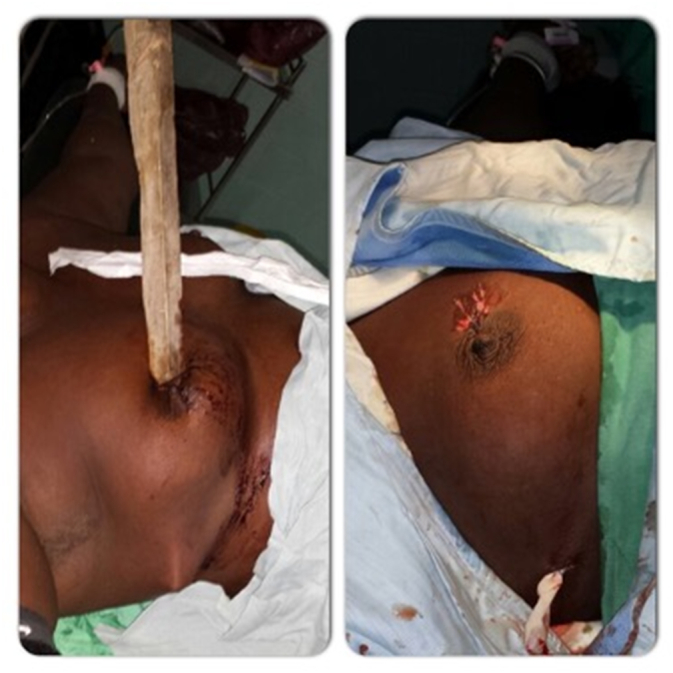
Before and after the surgical wound exploration of a parietal thoracic trauma following a fall, in our study population.

**Fig. 3:**
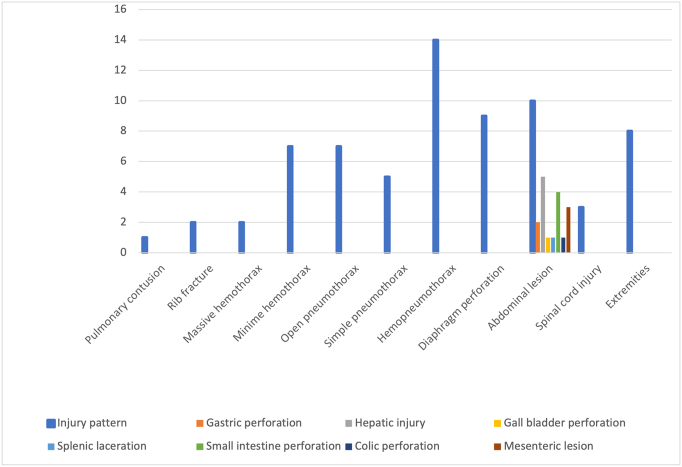
Injury pattern in our study population.

Table 2 summarizes the overall management of TT in our study. Pain management was done mostly with second step analgesics (*n* = 18, 51.4 %). There was an antibiotics prophylaxis for 33 patients (94.3 %). Conservative management was done for one patient. A surgical wound exploration was performed for a parietal injury following a fall (Fig. 2). Most of the patients underwent tube thoracostomy (*n* = 33, 94.3 %) and laparotomy was needed for 12 patients (34.3 %). From the laparotomy group, 2 cases were negative. The most common intraoperative findings at laparotomy were hepatic injury (*n* = 5, 14.3 %), followed by small intestine perforation (*n* = 4, 11.4 %), mesenteric lesion (n = 3, 8.6 %), gastric perforation (*n* = 2, 5.7 %), splenic laceration (*n* = 1, 2.9 %), gall bladder perforation (n = 1, 2.9 %) and colonic perforation (n = 1, 2.9 %) (Fig. 3).

**Table 2:** Overall care and outcome of the study patients.

	N	%
Pain control		
First step analgesics	16.0	46.0
Second step analgesics	18.0	51.0
Third step analgesics	1.0	3.0

Antibiotics		
Ceftriaxone	18.0	51.0
Ceftriaxone + Flagyl	13.0	37.0
Ampicillin	1.0	3.0
Cloxacillin	1.0	3.0
No antibiotics	2.0	6.0
Conservative management	1.0	2.9

Surgical care		
Wound exploration	1.0	2.9
Tube thoracostomy	32.0	91.4
Laparotomy	12.0	34.3
Spinal cord surgery	1.0	2.9
Vascular surgery	1.0	2.9

Laparotomy findings		
None	2.0	6.0
Gastric perforation	2.0	6.0
Hepatic injury	5.0	14.3
Gall bladder perforation	1.0	2.9
Splenic laceration	1.0	2.9
Small Intestinal perforation	4.0	11.4
Colic perforation	1.0	2.9
Mesenteric lesion	3.0	8.57

Estimated blood loss (ml)		
<750	15.0	42.8
750–1500	2.0	5.7
1500–2000	4.0	11.4
>2000	1.0	2.9

Transfusion		
Intraoperative	5.0	14.0
Postoperative	3.0	9.0

Complications		
Colo cutaneous fistula	1.0	2.9
Paraplegia	3.0	8.57

Survival		
Dead	2.0	5.7
Survivor	33.0	94.3

**Fig. 4:**
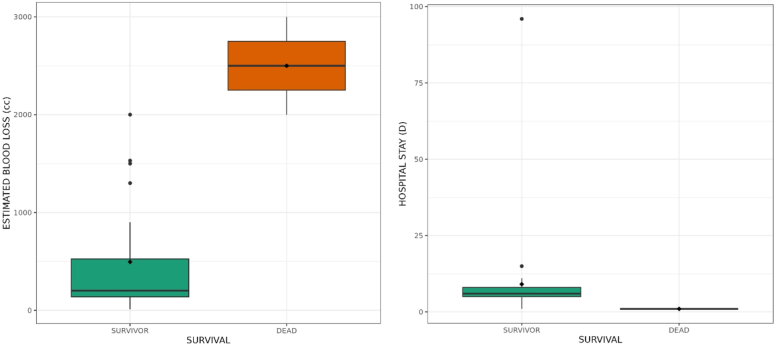
Relationship between estimated blood loss (in cc or ml), hospital stay (in days) and survival.

For patients undergoing surgery, the median EBL was 200 ml, range from 10 ml to 3000 ml, with 5 of them (14.3 %) losing >1500 ml. Transfusion was done for 8 patients (22.9 %) and the blood product that was used was packed red blood cells (PRBC). The maximum transfused volume was 3 units (1500 ml) of PRBC for one patient with an EBL >1500 ml. One patient with an EBL of approximately 2000 ml received only 2 units (1000 ml) of PRBC and one case with an EBL of 1500 ml did not have a transfusion (Table 3). We found a statistically significant association between the estimated blood loss and the survival. (*p* = 0.029) (Table 3 and Fig. 4).

**Table 3:** relationship between survival and parameters of thoracic trauma.

		Survival		n	p
		Survivor(n = 33)	Dead(n = 2)		
Age, mean (standard deviation)		37.5 (12.3)	34.0 (18.4)	35	0.83^a^
Hospital admission time (h)median [Q25–75]		6.00 [3.00; 12.0]	3.00 [2.00; 4.00]	32	0.35^b^
Hb, median [Q25–75]		11.3 [9.03; 12.9]	9.00 [7.00; 11.0]	28	0.79^b^
SBP at admission, median [Q25–75]		100 [92.5; 120]	120 [110; 130]	32	0.52^b^
Massive hemothorax, n(%)	Yes	30 (94 %)	2 (100 %)	32	1^c^
	No	2 (6.2 %)	0 (0 %)	2	–
EBL (ml), median [Q25–75]		200 [138; 525]	2500 [2250; 2750]	22	0.029^b^
Blood transfusion, n(%)	None	22 (79 %)	0 (0 %)	22	0.063^c^
	IntraOp	4 (14 %)	1 (50 %)	5	–
	Post-op	2 (7.1 %)	1 (50 %)	3	–
Associated lesion, n (%)	0	18 (56 %)	0 (0 %)	18	0.21^c^
	1	10 (31 %)	1 (50 %)	11	–
	2	4 (12 %)	1 (50 %)	5	–
Hospital stays (days)		6.0[5.0;8.0]	1[1.0;1.0]	35	0.029^b^

a: Welch, b: Mann-Whitney, c: Fisher.

h = hour, Hb = hemoglobin, SBP = systolic blood pressure, EBL = estimated blood loss, ml = millimeters.

## Postoperative data

Median hospital stay was 6 days, range from 1 to 96, days and median duration of the tube thoracostomy was 5 days, range 1 to 10 days. There were complications in 6 patients (17.1 %): three paraplegia (8.6 %), two death (5.7 %) and one enterocutaneous fistula (2.9 %). We had a survival rate of 94.3 % (*n* = 33) and a death rate of 5.7 % (*n* = 2).

## Discussion

This study reported a male to female ratio of 3:1. That male preponderance corroborates international data from both developed countries and LMIC. In Nigeria, Ekpe et al. found that ratio of 4:1, and in India, it varies from 2.4:1 (Walia et al) to 7:1 (Sharma et al) [[8], [9], [10]].

The population of our study had a median age of 40 years which is similar to India (37.2 years) and Nigeria (37.4 years) [8,11]. In our study the age group mainly concerned by TT is 31–50 years while in East China it was 31 to 70 years [12]. Battle et al. described extreme of age to be responsible for increased mortality in blunt chest trauma and Sikander N, Ahmad T, Shaikh A, et al. have made the same conclusion regarding age > 65 years [13,14]. In our investigation, there was no significant association between age and survival. However, the maximum age was 64 years in our study population, that corresponds to the data on the Haitian population, which is very young with a median age of 23 years in 2015 and a life expectancy of 63 in 2021 [4,15].

In our study, 5 patients (14.3 %) arrived within one hour, and 15 patients (45.7 %) arrived between 1 and 6 h. In contrast, the Nigerian study noted that 26 % presented beyond 24 h, accounting for 62.5 % of the mortality recorded [8]. The importance of the golden hour resides in the ability of the center to mobilize the needed resources to take care of the patient, which is generally not the case in the countries with limited resources [16]. In our study, one of the two patients who died, passed away despite being admitted within the golden hour because he sustained bilateral TT, associated abdominal injury, extremity injury (complete section of humeral artery). The second deceased patient was referred to the unit 5 h after the TT and presented a left TT with abdominal injury.

In our study the principal mechanism of TT is penetrating trauma (*n* = 33, 94.3 %) mostly caused by assaults (*n* = 30, 85.7 %), committed by strangers using firearms. That is similar in some point to Syria, were violence was the leading cause of TT, except that they showed a preponderance in blunt TT [17]. The findings of our study contrast with Pakistan where the principal mechanism of TT was blunt TT (56.9 %) caused by RTA and falls; they also had cases of blast injury [18]. India also showed a predominance of blunt TT (83.5 %) mainly caused by RTA (59.7 %) [11]. These differences testify to the current the climate of violence and insecurity in Haiti linked to firearms.

Clinical presentation varied according to the extent of the injuries. In our study, associated injuries were found in 45.7 % (*n* = 16) this is more than what is found in Nigeria (20.8 %) around the same period but similar to what was found in Germany (48 %) [8,19].

Concerning imaging, chest X-ray is the first line imaging in TT and may be the only imaging that can be performed when patient is unstable [20]. Haiti's healthcare system rest mainly on out-of-pockets payment, without health insurance, only 6 patients could afford an X-ray and one of them had an additional FAST [6].

Concerning management, most of our patients had a tube thoracostomy (*n* = 33, 94.3 %) with a median drainage duration of 5 days. But one third of our patients (*n* = 12, 34.3 %) needed an additional exploratory laparotomy. Similarly to our study, most of the patients in Germany (93 %) and India (75.3 %) were managed by chest tube insertion [11,19]. That corroborates the literature about management of TT. It is accepted that more that 90 % of patients with TT can be managed with needle decompression or tube thoracostomy while 5–10 % will require major surgery such as thoracotomy [10]. In East China, 33.2 % had a chest tube insertion with a duration of 1.2 days [12]. This difference in the duration of the chest tube in Haiti could be explained by the difficulties in monitoring the drainage, linked to the materiel used (mostly a non-graduated open recipient with water for sealing and without aspiration) and the lack of regular imaging for control.

The non-surgical expectative management in our study was done for one patient with blunt trauma following RTA. He sustained rib fractures and had a simple pneumothorax. That is in contrast to a center in India where most patients (55.43 %) were managed conservatively [9]. That same study in India showed a lower rate of laparotomy (16.45 %) compared to our study (*n* = 12, 34.3 %). This difference can be explained by the etiology of our TT, being mostly aggression with GSW.

In our study, we had a complication rate of 17.1 % (6 out of 35) while in Pakistan, the complication rate was 35.4 % [18]. Among our complications we had paraplegia following spinal cord injury and an enterocutaneous fistula consecutive to the laparotomy for a thoracoabdominal GSW. The latter had the longest hospital stay (96 days). Considering hospital stay, we noted that the median was 6 days. As the numbers of subjects compared were small, a non-parametric test was carried out (Mann-Whitney test), showing that the average rank of hospital stay is significantly different depending on survival (*p* = 0.029). In Germany the mean hospital stay varied from 31 to 34 days between groups, but they had patient admitted to ICU which was not the case in our study [19].

Concerning survival, the two patients with the highest ASA classification (ASA-IVE) survived. However, there was no statistical significance between ASA score and survival. Overall mortality in our study was 5.7 % and concerned two patients with penetrating TT. Mortality was higher in India (11 %) and concerned mostly blunt injuries [11]. In France, a study reported a mortality rate of 18.8 % in a group of penetrating TT [21].

In our study, the mortality concerned TT caused by GSW with abdominal associated injuries. Moreover, they had in common hypothermia at admission, diaphragmatic injury, liver injury with AAST liver trauma score ≥ grade 2, and an EBL ≥2000 ml. The staff was able to transfuse each one of them only 2 PRBC (equivalent to 1000 ml of blood). Haiti has one national transfusion center, not able to provide enough blood products for an estimated population of 12 million [22,23]. That brings to light the glaring insufficiency in blood products and the inability to perform massive transfusion. As the numbers of subjects compared were small, a non-parametric test was carried out (Mann-Whitney test), showing that the average rank of EBL (ml) is significantly different depending on Survival (*p* = 0.029). For many studies, the mortality rate was linked to the severity of trauma based on many score for example: Modified Early Warning Score (MEWS), Abbreviated Injury Score Thoracic (AIS thoracic), Injury Severity Score (ISS) [8,19]. Considering those scores, bilateral thoracic injury is a factor of severity due to the incapacity to ensure adequate ventilation-perfusion. In our investigation, bilateral thoracic injury was encountered in one of our two deaths, and the relationship was not statistically significant.

**Study limitations**: It is a monocentric investigation in a retrospective manner. This limited description of 35 cases, excluding children and patients admitted in the emergency ward, is mostly due to missing data linked to chart maintenance difficulties. For they are paper charts, stored in a shelter where has been placed the partially destroyed archive of HUEH after the earthquake. Among the missing data were the severity scores. We couldn't calculate those scores retrospectively with the available data. We will have to integrate those scores into a larger prospective study to allow standardization of data and better management.

## Conclusion

This is one of the rare studies in the Haitian environment that describes TT, all etiologies combined, their management and outcome. For that series, rather than road traffic accidents, assaults caused mainly by firearms were the chief cause of TT, testifying to the current climate of violence and insecurity in the country. Almost half of the patients sustained at least one associated injury. Although most of the TT cases were manage by tube thoracostomy, one third needed additional laparotomy. Estimated blood loss was associated with poorer patient outcomes, highlighting the need for optimizing the transfusion service in Haiti. The deaths recorded met the criteria for massive transfusion which could not be achieved. We highly recommend the use of trauma registry and severity scores when assessing patients with TT in the future. This study being a single-center observational investigation represents an open avenue for larger, multicentric studies to deepen the investigation and standardize the management of TT in Haiti.

## Meeting presentation

Abstract #: ASC20240232

Abstract title: Overview of thoracic trauma cases over a five-year period at a university hospital in Haiti

Session: 110-Clinical / Outcomes: Trauma / Critical Care Quickshot Session X.

Time: Thursday, February 8, 2024 @ 1:40 PM-3:40 PM (Eastern Time).

## CRediT authorship contribution statement

**Danielle Benjamin:** Conceptualization, Data curation, Formal analysis, Investigation, Methodology, Project administration, Resources, Software, Supervision, Validation, Visualization, Writing – original draft. **Patrick Charlorin:** Formal analysis, Methodology, Resources, Software, Writing – review & editing. **Gérald Jonacé:** Writing – review & editing, Resources. **Jude Milcé:** Resources, Supervision, Validation, Writing – review & editing.

## Funding

No funding received from any source.

## Declaration of competing interest

We do not have any conflict of interest.
